# TCF7L2 promotes anoikis resistance and metastasis of gastric cancer by transcriptionally activating PLAUR

**DOI:** 10.7150/ijbs.69933

**Published:** 2022-07-11

**Authors:** Tao Zhang, Bofang Wang, Fei Su, Baohong Gu, Lin Xiang, Lei Gao, Peng Zheng, Xue-mei Li, Hao Chen

**Affiliations:** 1 Department of oncology, The First Hospital of Lanzhou University, Lanzhou, Gansu, China.; 2 The second clinical medical college of Lanzhou university, Lanzhou , Gansu, China.; 3 Key laboratory of digestive system tumors, Lanzhou University Second Hospital, Lanzhou, Gansu, China.; 4 Cancer center, Lanzhou University Second Hospital, Lanzhou, Gansu, China.

**Keywords:** gastric cancer, urokinase-type plasminogen activator receptor, transcription Factor 7 Like 2, transcription, anoikis

## Abstract

Gastric cancer (GC) is the most common gastrointestinal malignant tumor, and distant metastasis is a critical factor in the prognosis of patients with GC. Understanding the mechanism of GC metastasis will help improve patient prognosis. Studies have confirmed that urokinase-type plasminogen activator receptor (PLAUR) promotes GC metastasis; however, its relationship with anoikis resistance and associated mechanisms remains unclear. In this study, we demonstrated that PLAUR promotes the anoikis resistance and metastasis of GC cells and identified transcription Factor 7 Like 2 (TCF7L2) as an important transcriptional regulator of PLAUR. We also revealed that TCF7L2 is highly expressed in GC and promotes the anoikis resistance and metastasis of GC cells. Moreover, we found that TCF7L2 transcription activates PLAUR. Finally, we confirmed that TCF7L2 is an independent risk factor for poor prognosis of patients with GC. Our results show that TCF7L2 and PLAUR are candidate targets for developing therapeutic strategies for GC metastasis.

## Introduction

Gastric cancer (GC) is the most common malignant tumor of the digestive system and the fifth most commonly diagnosed cancer worldwide 1. Compared to European and American countries, the morbidity and mortality of GC are higher in China 2. Despite the significantly improved prognosis of patients with GC with the development of novel drugs and advancement in therapeutic strategies, the long-term survival of patients with advanced GC remains dire. Distant metastasis is the primary influencing factor in the prognosis of patients with GC 3. Hence, it is necessary to understand the mechanism of GC metastasis and to identify metastasis-related molecular markers and therapeutic targets.

**Figure 1:**
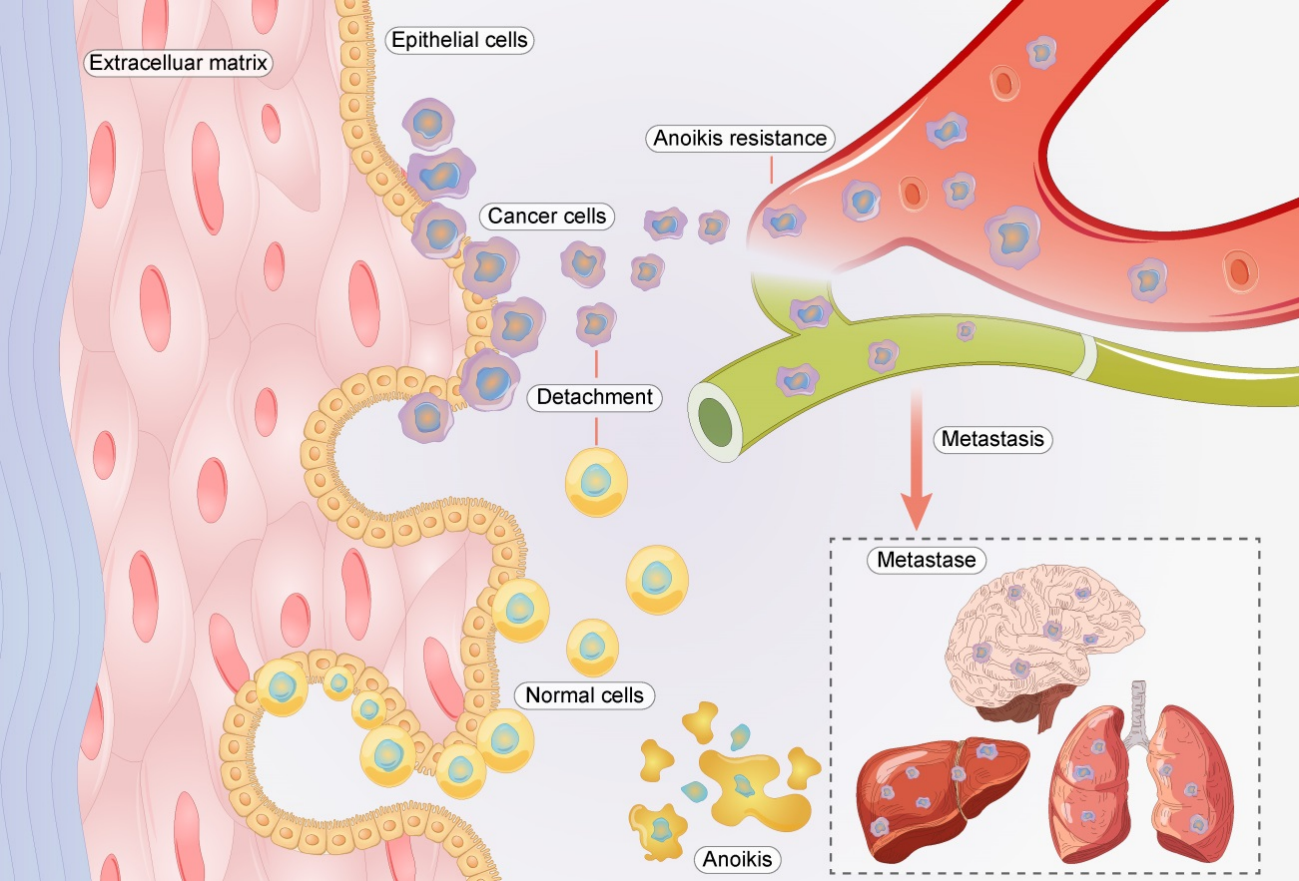
Relationship between anoikis resistance and metastasis of cancer cells.

“Anoikis” is cell death that occurs when cells lose interaction with surrounding stromal cells 6. Anoikis resistance is a hallmark event in the migration of cancer cells, as they must be able to survive after detachment from their supporting cells 4, 5. In cancer cells, anoikis resistance is regulated by multiple genes and signaling pathways 7, 8. Anoikis resistance plays a significant role in GC metastasis (**Figure 1**). For example, the nuclear MYH9-induced *CTNNB1* enhances the anoikis resistance of GC cells, ultimately promoting peritoneal metastasis of GC 9. L1CAM is upregulated in GC and is closely associated with the anoikis resistance of tumor cells 10. Briefly, cancer cells must overcome anoikis for successful metastasis.

As cancer malignancy progresses, the urokinase-type plasminogen activator receptor (uPAR or PLAUR) plays a key role, as it is not expressed in normal tissues but is strongly expressed in tumor tissues. The expression of PLAUR is linked to the invasion and migration of tumor cells 11-13. In tumor tissues, PLAUR can activate extracellular matrix proteolytic enzymes outside the cells by binding to urokinase-type plasminogen activator (uPA or PLAU), its ligand. This binding causes pathological changes in the extracellular matrix, ultimately creating favorable conditions for malignant behaviors such as the invasion and migration of tumor cells 14, 15. Numerous studies have demonstrated that PLAUR is upregulated in GC and is closely associated with cancer cell invasion and migration 16-18. However, its effect on the anoikis resistance of GC cells and its mechanisms remain unclear.

Transcription Factor 7 Like 2 (TCF7L2) is a member of the high-mobility group (HMG) family, a downstream effector of the Wnt/β-catenin signaling pathway, and a pivotal regulator of development and cell growth 19-22. TCF7L2 has a crucial role in various biological processes and functions of numerous organs and tissues, including liver, pancreatic islets, and adipose tissues 23, 24. Studies have suggested the close correlation of TCF7L2 with various somatic metabolic disorders. TCF7L2 leads to type 2 diabetes, primarily by affecting the metabolism and homeostasis of glucose 20, 25, 26. Recent studies have also confirmed its strong linkage to cancer. For example, the TCF7L2 gene polymorphism is closely associated with the onset of familial breast, colorectal, prostatic, and esophageal cancers 27-31. However, the correlation of TCF7L2 with GC as well as its effect on the anoikis resistance of GC cells has rarely been reported.

This study explored the association of PLAUR with the anoikis resistance of GC cells. TCF7L2 was identified as a transcriptional regulator of PLAUR. We confirmed that TCF7L2 activated the classic caspase-3/7 pathway in a PLAUR-dependent manner, thereby enhancing the anoikis resistance and migration of GC cells.

## Materials and Methods

### Clinical samples

Paired GC tissues and adjacent non-cancer tissues were acquired from the Lanzhou University Second Hospital. Cohort 1: PLAUR mRNA expression was detected in 15 pairs of fresh GC tissues and adjacent tissues, and the correlation between PLAUR and TCF7L2 protein expression was analyzed via immunofluorescence (IF) in these 15 GC tissues. Cohort 2: 121 pairs of GC tissues and adjacent non-cancer tissues were formalin-fixed and paraffin-embedded and then used to detect the expression of TCF7L2 protein in GC and adjacent tissues by immunohistochemistry (IHC). Cohort 3: 10 pairs of fresh GC tissues and adjacent non-cancer tissues were cryopreserved and used for quantitative analysis of TCF7L2 protein expression in GC and adjacent tissues by western blot. All enrolled patients signed informed consent, and procedures were approved by the Ethics Committee of Lanzhou University Second Hospital.

### Cell culture

GC cell lines MKN45, MGC-803, AGS, and HGC-27; gastric mucosal normal epithelial cell GES-1; and human embryonic kidney 293T (HEK293T) cells were purchased from Shanghai Fuheng Biotechnology (Shanghai, China). All cell lines were cultivated in Roswell Park Memorial Institute (RPMI) 1640 medium (Gibco, Waltham, MA, USA) supplemented with 10% fetal bovine serum (FBS; Gibco) and 1% penicillin streptomycin (Beyotime, Shanghai, China) in a 37 °C incubator with 5% CO_2_ under humidifying conditions.

### Cell transfection

Small hairpin RNA (shRNA) targeting PLAUR and TCF7L2, and negative control shRNA (sh-Ctrl) were synthesized by Compass Biotech (Quanzhou, China). The coding sequence of PLAUR or TCF7L2 was amplified and cloned into pCDH-CMV-MCS-EF1-copGFP plasmid (Compass Biotech) and packaged as lentivirus. Lentivirus was transfected into candidate GC cell lines. Positive cells were screened with puromycin (2 μg /mL) for 2 weeks, and the effect of knockdown and overexpression efficiency was measured via quantitative reverse transcription polymerase chain reaction (qRT-PCR) and western blot. The target sequences of PLAUR and TCF7L2 shRNAs are provided in **Table S1**.

### Construction of anoikis model

For the construction of the anoikis model, 1×10^6^ cells were suspended in ultra-low adsorption 6-well plates (Corning, NY, USA) and cultured for 24 h. Suspended cells were harvested and processed for subsequent experiments.

### qRT-PCR

Trizol reagent (Invitrogen, Grand Island, NY, USA) was used to extract total RNA from GC cells and tissue samples, according to the manufacturer's protocols. Total RNA was used for reverse transcription using PrimeScriptTM RT Reagent Kit (Takara, Shiga, Japan). Quantification was performed with SYBR Green PCR Master Mix (Takara) using Applied Biosystems ABI 7500 (Applied Biosystems, Waltham, MA, USA). GAPDH was used as an internal reference gene. The 2^-ΔΔCt^ method was utilized to quantify fold changes. All experiments were repeated in triplicate. Primer sequences are provided in **Table S2**.

### Western blot

Radio immunoprecipitation assay (RIPA, Beyotime, Shanghai, China) supplemented with protease inhibitors (Beyotime) was used to extract total protein from GC cells and tissue samples. Total protein was run on 8-12% SDS/PAGE gels and transferred onto polyvinylidene difluoride (PVDF) membranes (Millipore, Boston, MA, USA). Subsequently, membranes were blocked with 5% skim milk and incubated overnight at 4 °C with the primary antibodies. The next day, PVDF membranes were incubated for 2 h at room temperature with appropriate secondary antibodies, and ECL substrate was applied. Images were visualized using a Tianneng automatic chemiluminescence image processing system (TANO, Shanghai, China). Primary antibodies are presented in **Table S3**.

### Cell proliferation assay

The cell counting kit 8 (CCK-8, Yelasen, Shanghai, China) was used to quantify the proliferation ability of GC cells, according to the manufacturer's instructions. Cells in the sh-Ctrl group and shRNA group were seeded in 96-well plates (Corning) at 1×10^4^ cells/well. After 24 h, 48 h, and 72 h, 10 μL of CCK8 solution was mixed with the cells and incubated for 2 h at 37 °C to assess proliferation activity. The absorbance was measured at 450 nm using a BioTek microplate reader (BioTek, Winooski, VT).

### 5-ethynyl-2'-deoxyuridine (EdU) assay

An EdU assay was performed using an EdU assay kit (RiboBio, Guangzhou, China). Cells were incubated with 50 μm Edu solution at room temperature for 6-8 h. Subsequently, cells were fixed with 4% neutral paraformaldehyde at room temperature for 30 min. Cells were permeabilized with PBS containing 0.5% Triton X-100 for 20 min and then washed three times with PBS. Cells were stained with Apollo staining reagent (KeyGEN, Nanjing, China) for 20 min and washed three times with NaCl/PI. Cell nuclei were stained with 4',6-diamidino-2-phenylindole (DAPI, KeyGEN) and visualized under a microscope.

### Transwell assay

Transwell chambers (Corning) were used to measure cell migration ability. Cells were suspended in RPMI 1640 medium without FBS, and 1×10^4^ cells were seeded into the upper chamber of the transwell chamber. Subsequently, 800 μL of RPMI 1640 medium with 10% FBS was added into the lower chamber. After 24 h, cells that had migrated to the lower surface of membranes were fixed with 4% paraformaldehyde for 20 min and stained with 0.1% crystal violet for 20 min. The average number of migrating cells was determined by six randomly picked fields of vision under the microscope.

### Wound healing assay

The wound healing assay was processed using 6-well plates (Corning). The cells of different groups were seeded into 6-well plates and cultivated until the cells were 90% confluent. A scratch was made with a pipettor tip, the detached cells were washed off with PBS, and then cells were cultured in medium without FBS for 24 h. The wound widths were assessed at 0 h and 24 h under a light microscope, and ImageJ software (ImageJ Software Inc., MD, USA) was used to assess the relative area of wound closure.

### Cell apoptosis assay

Flow cytometry was used for apoptosis assays. The AnnexinV-FITC apoptosis detection kit (Yelasen, Shanghai, China) was used to measure the apoptosis rates of adherent and suspension cells. Cells were washed twice with PBS buffer, 300 μL of binding buffer was added to tubes, and stained by 5 μL Annexin V-FITC and 10 μL PI in the dark for 15 min at room temperature. The percentage of cell apoptosis was measured via flow cytometry (Beckman Cytoflex, California, CA, USA).

### IF

Cells or tissues on slides were fixed with paraformaldehyde (4%) and permeabilized with 0.01% Triton X-100 for 15 min. The slides were incubated with primary antibodies anti-PLAUR (1:200, Santa Cruz, sc-13522) or anti-TCF7L2 (1:100, Bioss, bsm-52543R), overnight in a humidified chamber at 37 °C in the dark. Subsequently, cell nuclei were stained with DAPI. The fluorescence distribution and intensity of cells and tissues were analyzed using a Zeiss LSM 880 laser microscope (Carl Zeiss AG, Oberkochen, Germany).

### DNA pull-down assay

The DNA-pull-down assay was performed using the Bes5004 DNA pull-down kit (BersinBio, Guangzhou, China), according to the manufacturer's instructions. First, the *PLAUR* promoter sequence was searched for in the NCBI (**Table S4**). PCR products were labeled with biotin using a universal biotinylated primer. The gel recovery kit (QIAGEN, Venlo, Netherlands) was used to isolate a labeled probe from agarose gels. Lysis buffer was used to extract MKN45 and 293T cell total protein, and a BCA kit (Solarbio, Beijing, China) was used to measure the protein concentration. One part of the protein was marked as “Input,” and the other part was incubated with the probe to form a protein-DNA-magnetic bead complex. The targeted protein was marked as “Target” after it was eluted and then run on SDS-PAGE. Differential bands were analyzed by silver staining. The fractions were analyzed using nanospray liquid chromatography tandem mass spectrometry (LC-MS/MS) on a Thermo Scientific QE HF (Thermo Fisher Scientific, MA, USA).

### Chromatin-immunoprecipitation (ChIP)

ChIP assays were executed using the SimpleChIP® Enzymatic Chromatin IP Kit (CST, Massachusetts, MA, USA). The cells were fixed by 1% paraformaldehyde for 10 min, then 0.125 M glycine was added and the mixture was placed at room temperature for 5 min to terminate the DNA-protein crosslinking. SDS lysis buffer (with protease inhibitors) was added to the cells, and an ultrasonic fragmentation device was used to generate chromatin fragments. A portion of the products was marked as “Input.” The remaining lysates were incubated with TCF7L2 antibody (Abcam, Cambridge, UK) and Protein G magnetic beads to form a DNA-antibody-magnetic beads complex, and the DNA was marked as “Target” after elution and purification. Rabbit IgG (CST, Massachusetts, MA, USA) served as a negative control. The final purified DNA fragment was analyzed via qRT-PCR. Primers of qPCR for the *PLAUR* promoter binding site are presented in **Table S5**.

### Luciferase activity assay

Luciferase activity assay was performed using the Luciferase Detection Kit (Promega, Madison, WI, USA), according to the manufacturer's instruction. First, The WT and mutated PLAUR UTR were cloned into PGL4.11-Basic Vector (Promega), producing the vectors PGL4.11-PLAUR-WT-promoter-luc and PGL4.11-PLAUR-mut-promoter-luc MKN45, and 293T cells were co-transfected with different plasmids for 48 h. Finally, the Promega dual-luciferase system (Promega) was used for detection.

### Co-immunoprecipitation (co-IP) assay

Co-IP assay was performed as described previously 32. Lysates were incubated with Flag affinity beads (Sigma-Aldrich, MA, USA). The interacting proteins were detected via western blot.

### Xenograft assay

MKN45 cells were transfected with shRNA against PLAUR, TCF7L2 and the corresponding scramble interference by lentiviral infection. Each plasmid had a luciferase label. Cells transfected with sh-Ctrl and shRNAs (sh-PLAUR or sh-TCF7L2) 1:1 were mixed with PBS and Matrigel suspension reagent (BD Biosystems, San Jose, CA, USA). Briefly, 1×10^6^ cells were injected subcutaneously into female BALB/c nude mice aged 6-8 weeks (Hangzhou Ziyuan Laboratory Animal Science and Technology). Tumor volumes were measured every 4 days and calculated by the formula (length × width^2^)/2. After 24 days, mice were sacrificed by deep anesthesia with ethyl ether. Tumor tissues were harvested for subsequent experiments. The lung metastasis xenograft model was established by injecting 1×10^6^ cells into female BALB/c nude mice aged 6-8 weeks through the tail vein. The bioluminescence images were acquired by the IVIS spectrum imaging system (PerkinElmer, Massachusetts, USA). Lastly, mice were sacrificed after 28 days, and lung tissues were harvested for subsequent experiments. Animal experiments were reviewed and approved by the Animal Use Ethics Committee of Lanzhou University Second Hospital. All animals were cared for in accordance with the Guide for the “Care and Use of Laboratory Animals”.

### Hematoxylin and eosin (H&E) staining and IHC

H&E staining was performed on 5 μm paraffin sections using a standard H&E staining protocol 33. Tissue samples were fixed, embedded, cut into slices, dewaxed, antigen retrieved, incubated with primary antibody, incubated with secondary antibody, DAB stained, hematoxylin stained, and alcohol denatured. The primary antibodies were anti-PLAUR (1:200, Santa Cruz, sc-13522), anti-TCF7L2 (1:100, CST, #2569), and anti-Ki67 (1:100, Proteintech, 27309-1-AP). Two experienced pathologists independently reviewed all sections. The IHC score was determined using modified Histo-score (H-score) 34. The staining intensity and percentage of positive cells were scored as described previously 35. The final immunoreactive score was calculated as the staining intensity score × the percentage of positive cells. The expression level of TCF7L2 was considered “low expression” if the immunoreactive score was 0-4 and “high expression” if the score was 5-9.

### Bioinformatics analyses

Stomach adenocarcinoma (STAD) gene mutation data and RNA-seq data were obtained from the TCGA database. GenVisR 36 data package was used to construct and draw the “waterfall map” of STAD gene mutation. UCSC Genome Browse (http://genome.ucsc.edu) 37 and PROMO database (http://alggen.lsi.upc.es/home.html) 38 were used to search for PLAUR promoter sequences and analyze transcription factors that regulate PLAUR. The Pearson correlation between the expression of PLAUR and its transcription factor TCF7L2 was calculated using the “ggstatsplot” and “ggplot2” packages. The JASPAR database (https://jaspar.genereg.net/) 39 was used to identify the binding site of transcription factor TCF7L2 to the PLAUR promoter. Expression profiles and protein level of TCF7L2 in STAD were acquired from GEPIA (http://gepia.cancer-pku.cn/) 40 and HPA (https://www.proteinatlas.org) 41 databases. The survival of patients with GC against TCF7L2 expression was estimated using a Kaplan-Meier plotter (http://www.kmplot.com/) 42.

### Statistical analysis

Functional analysis was performed using the SPSS software package (version 24.0, IBM SPSS, IL, USA) and GraphPad Prism (version 8.0, GraphPad Software, CA, USA). We used Student's t-test to examine the difference in means between two groups, and two-tailed ANOVA to examine the difference between multiple groups. A Chi-square test was used for correlation analysis between gene expression and clinicopathological features. The Kaplan-Meier method was used to test the overall survival between different groups. Cox regression analysis was used to analyze the influencing factors affecting the prognosis of patients. A *P* value < 0.05 was considered a statistically significant difference.

## Results

### PLAUR promotes the proliferation, anoikis resistance, and migration of GC cells

**Figure 2:**
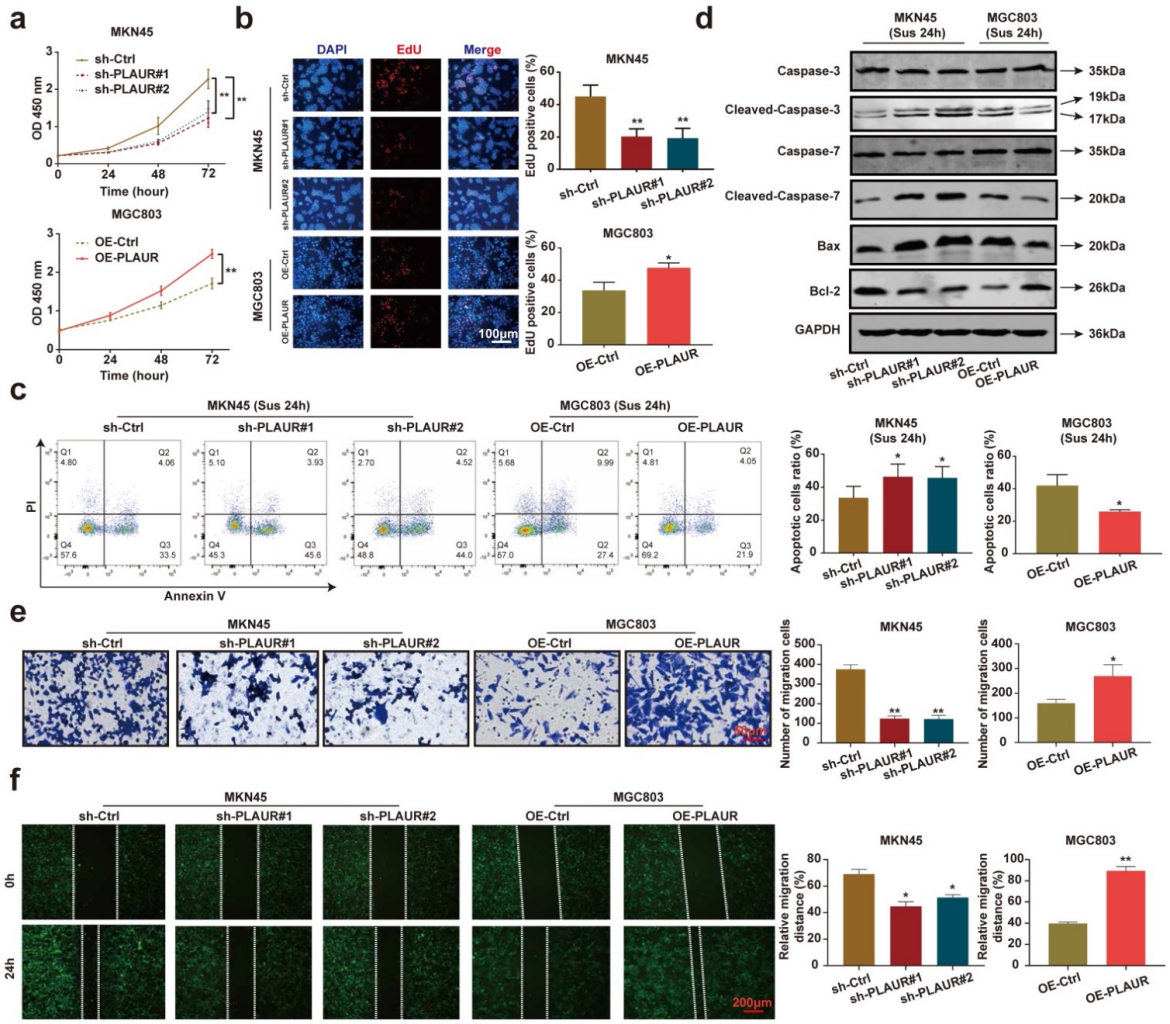
** PLAUR promotes the malignant phenotype of GC cells. a-b** Effects of PLAUR knockdown or overexpression on the proliferation of GC cells, measured by CCK-8 **(a)** and EdU assay **(b)**. **c** After GC cells were suspended for 24h, the effects of PLAUR knockdown or overexpression on the anoikis resistance of GC cells, analyzed via flow cytometry.** d** Changes in apoptosis-related proteins after PLAUR knockdown or overexpression were analyzed via western blot in GC cells suspended for 24 h. **e-f** Effects of PLAUR knockdown or overexpression on the migration of GC cells, evaluated by transwell **(e)** and wound healing assay** (f)**. Compared with the corresponding control group, * *P* < 0.05, ** *P* < 0.001.

To analyze the effects of PLAUR on the malignant behavior of GC cells, we quantified its expression in normal gastric epithelial cells (GES-1) and GC cell lines (HGC27, MGC803, AGS, and MKN45). The expression of PLAUR was highest in MKN45 cells and lowest in MGC803 cells (**Supplementary Figure 1**). Accordingly, these two cell lines were used to construct stable PLAUR knockdown or overexpressing cells. qRT-PCR and western blot were used to confirm the knockdown or overexpression (**Supplementary Figures 2**). Afterwards, we assayed the effects of PLAUR on GC cell proliferation via CCK-8 and EdU assays. The results showed that the knockdown of PLAUR inhibited the proliferation of MKN45 cells, while its overexpression promoted the proliferation of MGC803 cells (**Figure 2a-b**). To mimic the anoikis of GC cells, we used ultra-low attachment 6-well plates to suspend the cells for 24 h. Thereafter, the impact of PLAUR on the anoikis resistance of GC cells was analyzed via flow cytometry. Following a 24 h suspension of cells, the knockdown of PLAUR promoted the apoptosis of MKN45 cells, whereas its overexpression inhibited the apoptosis of MGC803 cells (**Figure 2c**). Meanwhile, we used western blot to detect the apoptosis-related protein expression in the GC cells that had been suspended for 24 h. As shown in **Figure 2d**, after cell suspension for 24 h, the knockdown of PLAUR promoted the expression of cleaved-caspase-3/7 and Bax in the MKN45 cells, while inhibiting the expression of Bcl-2. In contrast, the overexpression of PLAUR inhibited cleaved-caspase-3/7 and Bax expression in MGC803 cells, while promoting the expression of Bcl-2. Finally, transwell and wound healing assays were employed to analyze the correlation of PLAUR with the migration of GC cells. The knockdown of PLAUR inhibited the migration of MKN45 cells, while its overexpression promoted the migration of MGC803 cells (**Figure 2e-f**). Overall, PLAUR promoted the proliferation, anoikis resistance, and migration of GC cells.

### PLAUR promotes tumor growth and metastasis *in vivo*

**Figure 3:**
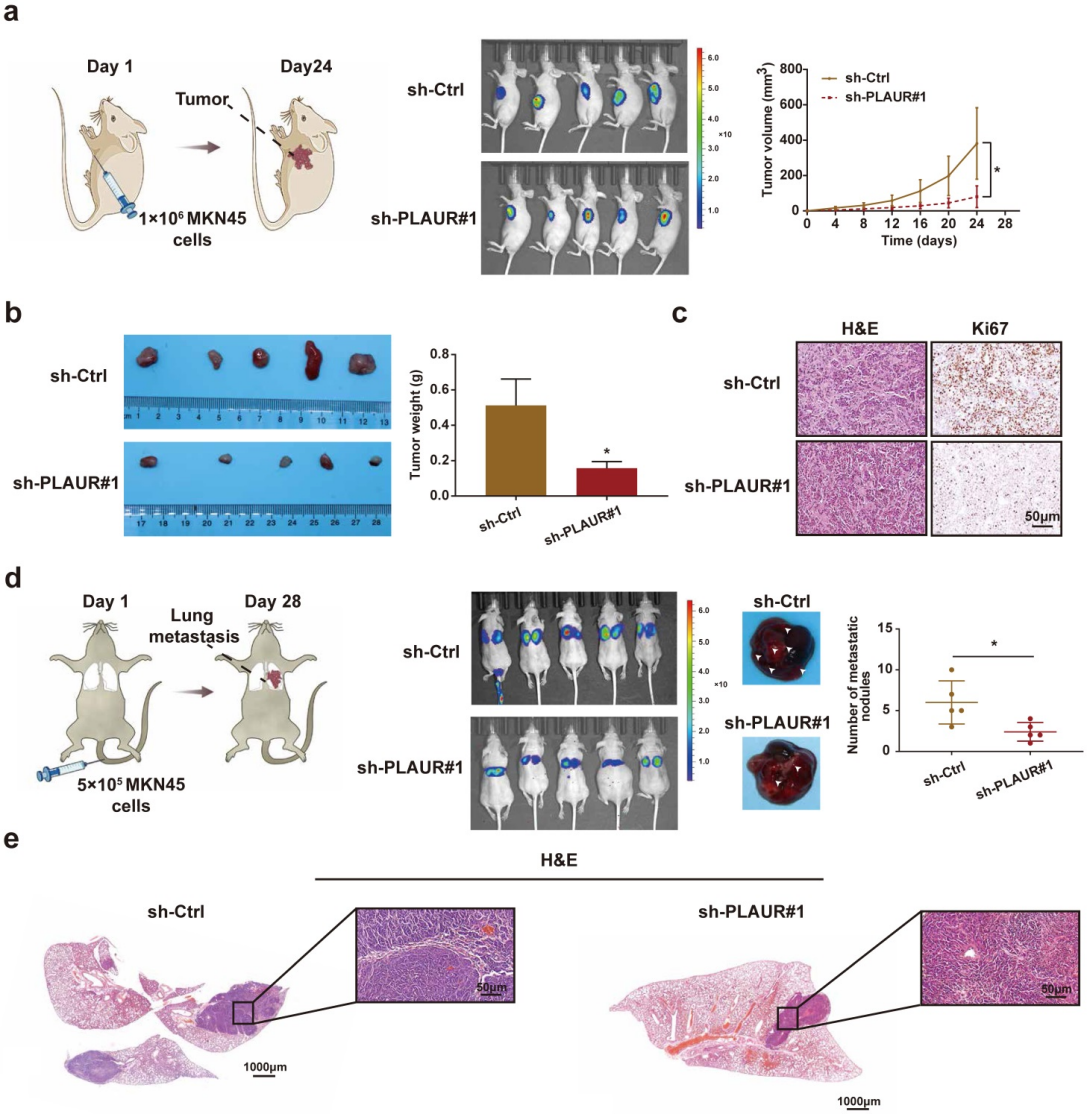
** PLAUR promotes the GC process *in vivo*. a** Knockdown of PLAUR inhibited the growth of subcutaneous xenograft tumors in nude mice. The bioluminescence images of the subcutaneous tumors were displayed in a small animal* in vivo* imaging system, and the tumor growth curve for 24 days was measured. **b** Gross specimen and average weight of the tumor after sacrifice. Knockdown of PLAUR inhibited the volume and weight of subcutaneous xenograft tumors in nude mice. **c** Histological characteristics and Ki67 expression of the tumor were examined via H&E staining and IHC, respectively. The knockdown of PLAUR inhibited the Ki67 expression of subcutaneous xenograft tumors in nude mice. **d** Knockdown of PLAUR inhibited lung metastasis in nude mice. The bioluminescence image of lung metastases and the count of lung metastatic nodules are displayed, 28 days after tail veins were injected with GC cells. The white arrows represent the lung metastasis nodules. **e** Lung metastasis tumors were confirmed via H&E staining. Compared with the control group, * *P*<0.001.

To further confirm our *in vitro* observations, we explored whether PLAUR regulates the growth and metastasis of tumor cells *in vivo*. Initially, we established a nude mouse model of subcutaneous tumors by subcutaneously injecting MKN45 cells with sh-Ctrl or sh-PLAUR#1. We observed the changes in subcutaneous tumor volume of nude mice and plotted corresponding growth curves. The tumors were also confirmed by small animal live imaging on the 24^th^ day after injection. As expected, PLAUR knockdown significantly delayed *in vivo* tumor growth in nude mice (**Figure 3a**). Tumors harvested from the sacrificed animals were weighed, and significantly heavier tumors were observed in nude mice in the sh-Ctrl group than in the sh-PLAUR#1 group (**Figure 3b**). Additionally, subcutaneous tumors were identified via H&E staining (**Figure 3c**). IHC analysis revealed that PLAUR knockdown inhibited the expression of Ki67 in tumor tissues (**Figure 3c**). Subsequently, we established a nude mouse model of lung metastasis through tail vein injection of MKN45 cells, either with sh-Ctrl or sh-PLAUR#1. On the 28^th^ day following injection, mice in the sh-Ctrl group exhibited significantly higher lung tumor burden than those in the sh-PLAUR#1 group (**Figure 3d**). After euthanizing and dissecting mice, the PLAUR knockdown reduced the number of nodules on the lung surfaces (**Figure 3d**). As shown in **Figure 3e**, H&E staining was also employed to identify lung metastases. Collectively, the above results confirmed that PLAUR facilitates tumor growth and metastasis *in vivo*.

### TCF7L2 is a potential transcriptional regulator of *PLAUR*

As described earlier, PLAUR was upregulated in GC, which promoted the malignancy of tumor cells, including anoikis resistance. Afterwards, we explored the reasons for PLAUR upregulation in GC. Initially, we examined PLAUR mRNA expression in 15 pairs of GC and adjacent tissues (cohort 1). The results indicated considerably higher PLAUR mRNA content in the GC tissues than in the adjacent tissues (**Supplementary Figure 3**). Additionally, we ruled out the possibility of PLAUR upregulation in GC caused by* PLAUR* mutations using a bioinformatics approach (**Supplementary Figure 4**). Meanwhile, considering the major role of abnormal transcriptional regulation in carcinogenesis and development, we speculated that abnormal transcription leads to upregulated PLAUR expression in GC.

**Figure 4:**
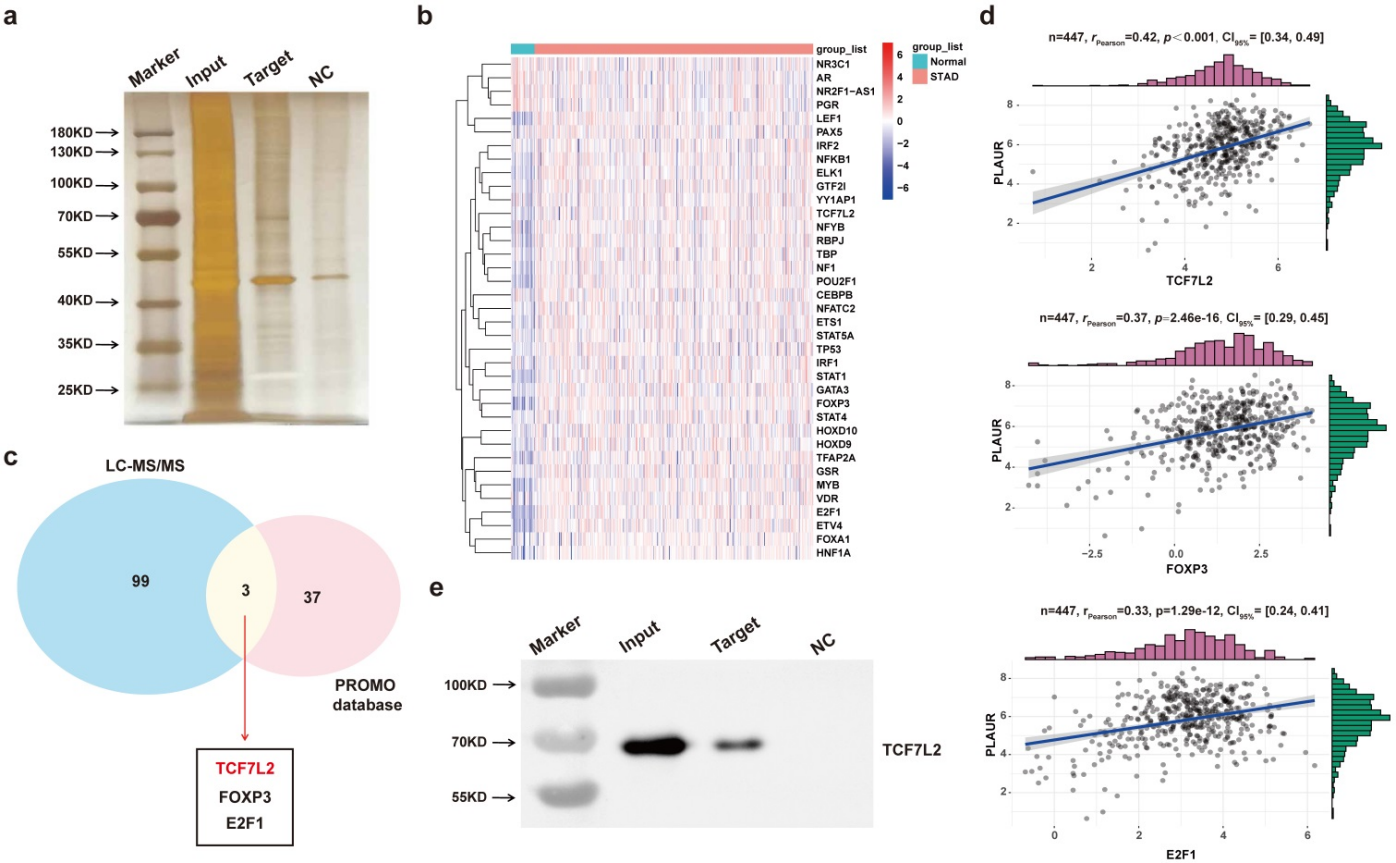
** TCF7L2 is a potential transcriptional regulator of* PLAUR*. a** Silver staining of PLAUR binding protein identified in DNA pulldown.** b** Heat map of expression of transcription factors of *PLAUR i*n GC samples and normal samples. **c** TCF7L2, FOXP3, and E2F1 were identified as transcription factors of *PLAUR* through LC-MS/MS, combined with the screening results of the PROMO database. **d** Expression correlations of TCF7L2, FOXP3, E2F1, and PLAUR were analyzed based on TCGA-seq data. TCF7L2 has the highest correlation with PLAUR expression. **e** TCF7L2 protein was confirmed to be present in the pull-down product by western blot.

To find major transcription factors capable of regulating PLAUR expression, we first determined the proteins that bind to the *PLAUR* promoter in MKN45 cells, by DNA pull-down assay. As shown in **Figure 4a**, many DNA-protein complex bands were found on silver-stained polyacrylamide gels, some of which were absent in the control reaction. We further identified the aforementioned proteins through LC-MS/MS (**Table S6**). Subsequently, PROMO program was used to discover the potential transcription factors that can regulate PLAUR expression, and TCGA-STAD-seq data were utilized to draw an expression heatmap of these transcription factors in GC (**Figure 4b**). Notably, three transcription factors, namely TCF7L2, FOXP3, and E2F1, overlapped in the LC-MS/MS and PROMO searches (**Figure 4c**). Based on the TCGA-STAD-seq data, we analyzed the correlation of PLAUR expression with these transcription factors in GC. As shown in **Figure 4d**, TCF7L2 exhibited the highest correlation with PLAUR expression (*r* = 0.42, *P* < 0.001). Finally, the expression of TCF7L2 protein in the pull-down product was detected via western blot (**Figure 4e**). Further analysis, aided by online bioinformatics tools, revealed that TCF7L2 was highly expressed in GC, localized primarily in cell nuclei, and was associated with poor patient prognosis (**Supplementary Figure 5**). Our data suggested that the transcription factor TCF7L2 plays an oncogenic role in GC. Hence, we focused on exploring the regulatory relationship between TCF7L2 and PLAUR.

### TCF7L2 transcriptionally upregulates PLAUR expression in GC

**Figure 5:**
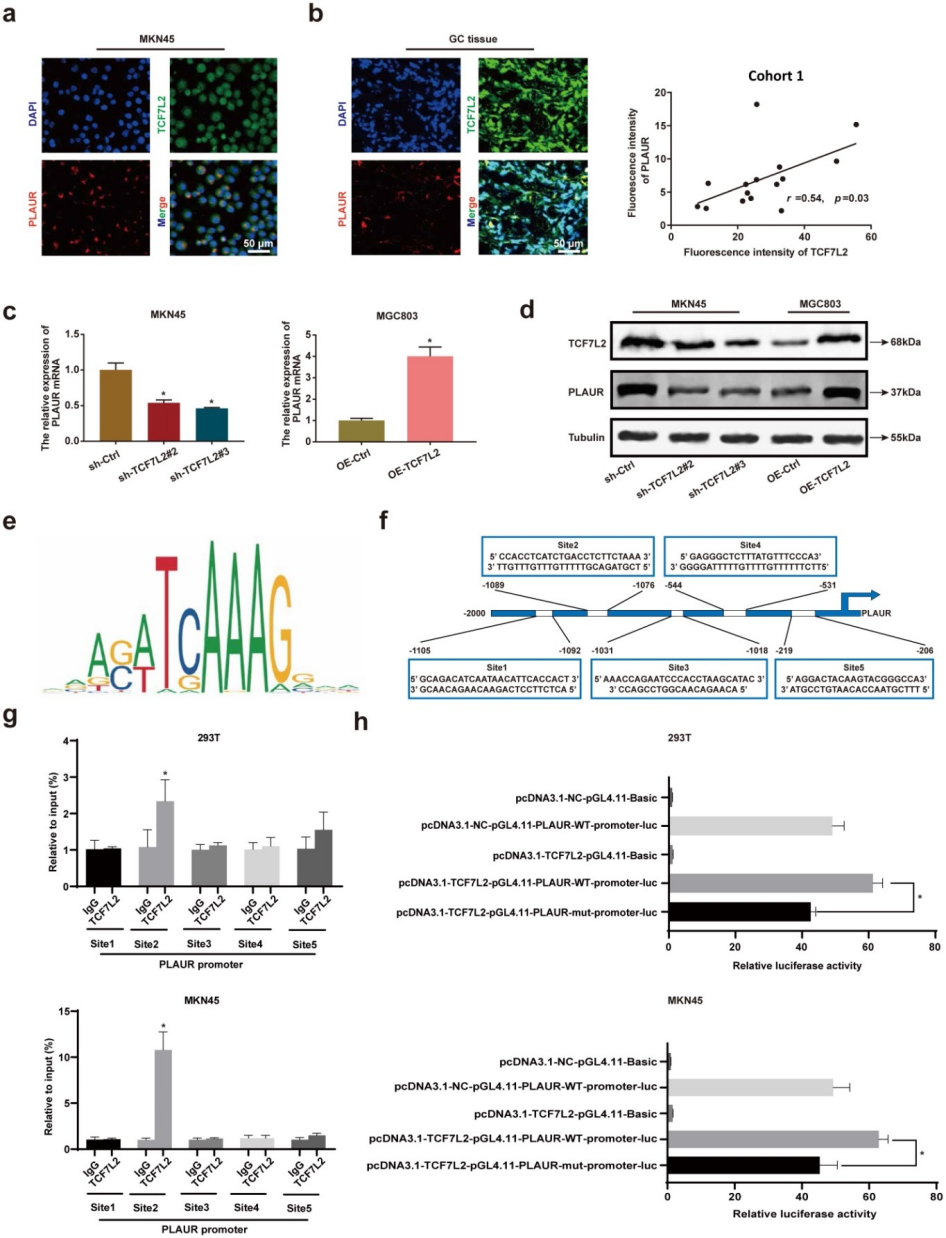
** TCF7L2 promotes transcription of *PLAUR* in GC. a** Representative image of the co-localization of TCF7L2 protein (green) and PLAUR (red) in MKN45 cell lines observed by confocal microscopy. **b** Representative image of the co-localization of TCF7L2 protein (green) and PLAUR (red) in 15 GC tissues (cohort 1) observed via confocal microscopy; the expression of TCF7L2 and PLAUR was positively correlated via fluorescence quantitative analysis.** c-d** TCF7L2 has a positive regulatory effect on the expression of PLAUR in GC cells. The effects of TCF7L2 silencing on PLAUR mRNA or protein expression in GC cells as detected by RT-qPCR **(c)** and western blot **(d)**, respectively. **e** Motif of the TCF7L2 binding site in the *PLAUR* promoter, predicted through the JASPAR dataset. **f** Schematic of the TCF7L2 binding site on the* PLAUR* promoter. **g** ChIP-qPCR analysis of TCF7L2 binding to the *PLAUR* promoter in MKN45 and 293T cell lines. The second position was the most important binding site. **e** Luciferase activity was determined after mutation of the second TCF7L2 site in the *PLAUR* promoter in MKN45 and 293T cell lines. The luciferase activity of the wild-type *PLAUR* promoter was significantly higher than that of mutant *PLAUR* promoter. Compared with the corresponding control group, * *P* < 0.001.

To further assess the regulation of PLAUR expression by TCF7L2 at the transcriptional level, we studied the localization of TCF7L2 and PLAUR proteins in MKN45 cells and GC tissues by IF staining, with GC tissues from cohort 1. TCF7L2 protein was detected at varying levels in the cytoplasm and nucleus of both MNK45 cells and GC tissues, whereas PLAUR protein was observed only in the cytoplasm (**Figures 5a-b**). Through fluorescence intensity, we found that the expression of TCF7L2 and PLAUR were positively correlated in 15 GC tissues (*r* = 0.54, *P* < 0.03; **Figure 5b**). Notably, we observed the co-localization of TCF7L2 and PLAUR proteins in the cytoplasm of GC cells through confocal laser microscopy. Hence, a co-IP assay was employed to further analyze whether there was a protein-protein interaction between TCF7L2 and PLAUR which could regulate the abnormal PLAUR expression in GC. The results revealed an absence of direct interaction between TCF7L2 and PLAUR protein (**Supplementary Figure 6**). Cell nuclei are considered the main site where transcription events occur in organisms. Overall, it can be deduced that TCF7L2 transcriptionally regulates PLAUR expression.

To validate the regulatory effect of TCF7L2 on PLAUR expression, we suppressed TCF7L2 expression in MKN45 cells and overexpressed TCF7L2 in MGC803 cells. The knockdown or overexpression was verified by qRT-PCR and western blot (**Supplementary Figure 7**). Afterwards, the effects on PLAUR expression were analyzed. The knockdown of TCF7L2 greatly inhibited PLAUR expression in MKN45 cells, whereas its overexpression elevated PLAUR in MGC803 cells (**Figure 5c-d**). Accordingly, we confirmed that TCF7L2 regulates PLAUR expression in GC.

To assess whether TCF7L2 protein can directly bind to the *PLAUR* promoter, we identified multiple binding sites using the JASPAR database (**Figure 5e; Table S7**). For the top 5 binding sites on the JASPAR database (**Figure 5f**), we performed ChIP analysis in MKN45 and 293T cells. As shown in **Figure 5g**, the second site (-1089 to -1076) in the *PLAUR* promoter region was the primary binding site of TCF7L2. Subsequently, to further validate the transcriptional activation effect of TCF7L2 on* PLAUR*, we again conducted a dual luciferase reporter assay using MKN45 and 293T cells. Based on the ChIP-qPCR results, we mutated the second site in the *PLAUR* promoter region where TCF7L2 primarily binds and then examined the luciferase activity. The luciferase activity of the wild-type *PLAUR* promoter was significantly higher than that of the mutant *PLAUR* promoter (**Figure 5h**). This suggested that the transcription factor TCF7L2 has a positive regulatory relationship with its downstream target gene, *PLAUR*. In summary, TCF7L2 promotes PLAUR expression through transcriptional upregulation.

### TCF7L2 promotes GC carcinogenesis *in vivo* and *in vitro*

**Figure 6:**
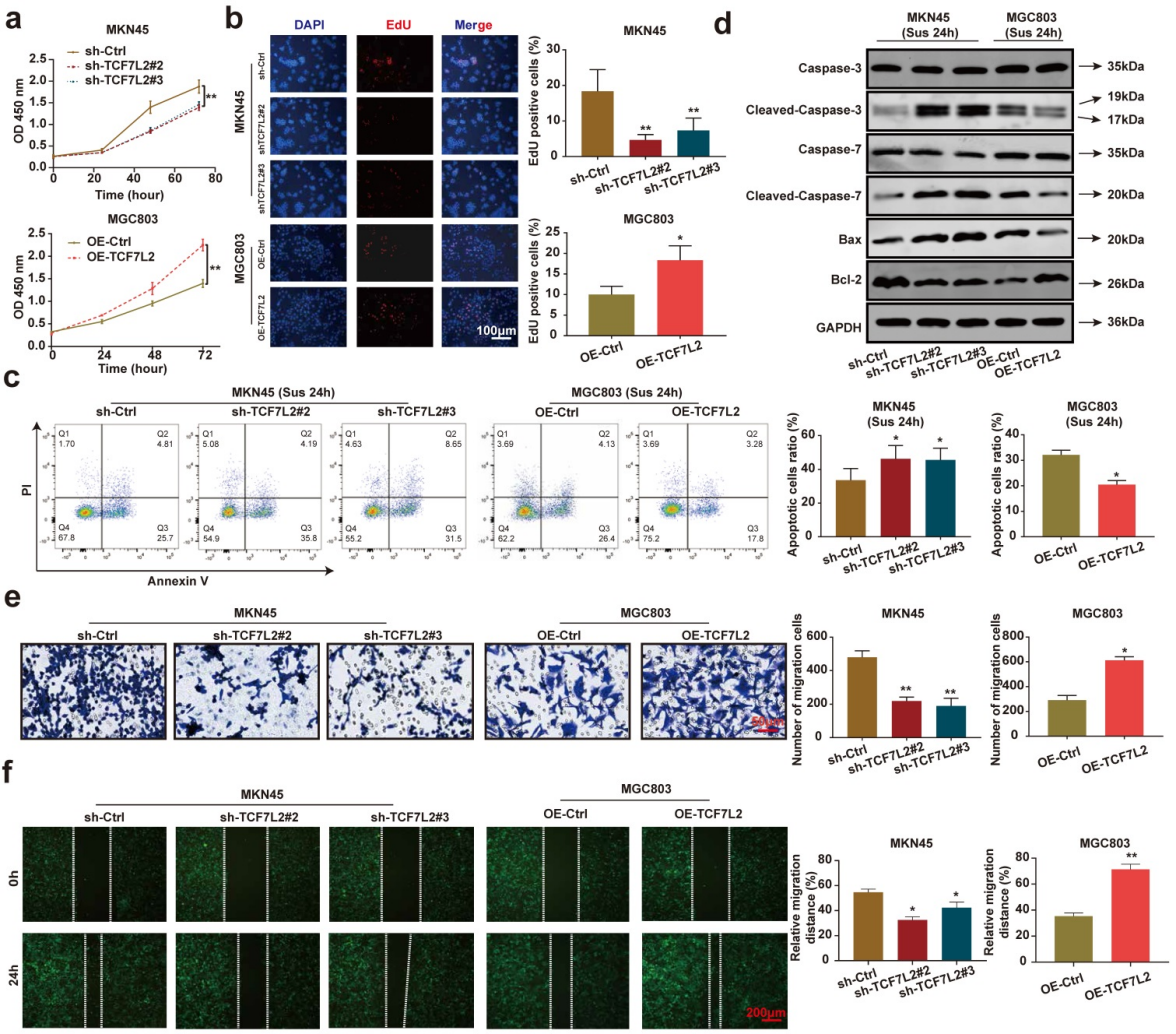
** TCF7L2 promotes the malignant phenotype of GC cells. a-b** Effects of TCF7L2 knockdown or overexpression on the proliferation of GC cells measured by CCK-8 **(a)** and EdU assay **(b)**. **c** After GC cells were suspended for 24h, the effects of TCF7L2 knockdown or overexpression on the anoikis resistance of GC cells were analyzed via flow cytometry.** d** Changes in apoptosis-related proteins after TCF7L2 knockdown or overexpression were analyzed via western blot in GC cells suspended for 24 h. **e-f** Effects of TCF7L2 knockdown or overexpression on the migration of GC cells as evaluated by transwell **(e)** and wound healing assay** (f)**. Compared with the corresponding control group, * *P* < 0.05, ** *P* < 0.001.

We focused on investigating the effects of TCF7L2 on carcinogenesis *in vitro* and *in vivo*. As shown in **Figures 6a-b**, the knockdown of TCF7L2 inhibited the proliferation of MKN45 cells, whereas its overexpression promoted the proliferation of MGC803 cells. After 24 h suspension culture, the knockdown of TCF7L2 promoted the apoptosis of MKN45 cells, whereas its overexpression inhibited the apoptosis of MGC803 cells (**Figure 6c**). Western blot results showed that after suspension culture for 24 h, the knockdown of TCF7L2 promoted the expression of cleaved-caspase-3/7 and Bax in MKN45 cells, while inhibiting Bcl-2 expression. In contrast, the overexpression of TCF7L2 inhibited the expression of cleaved-caspase-3/7 and Bax in MGC803 cells, while promoting Bcl-2 expression (**Figure 6d**). As expected, the knockdown of TCF7L2 inhibited the migration of MKN45 cells, whereas its overexpression promoted the migration of MGC803 cells (**Figure 6e-f**). Briefly, our data indicated that TCF7L2 affects the proliferation, anoikis resistance, and migration of GC cells.

**Figure 7:**
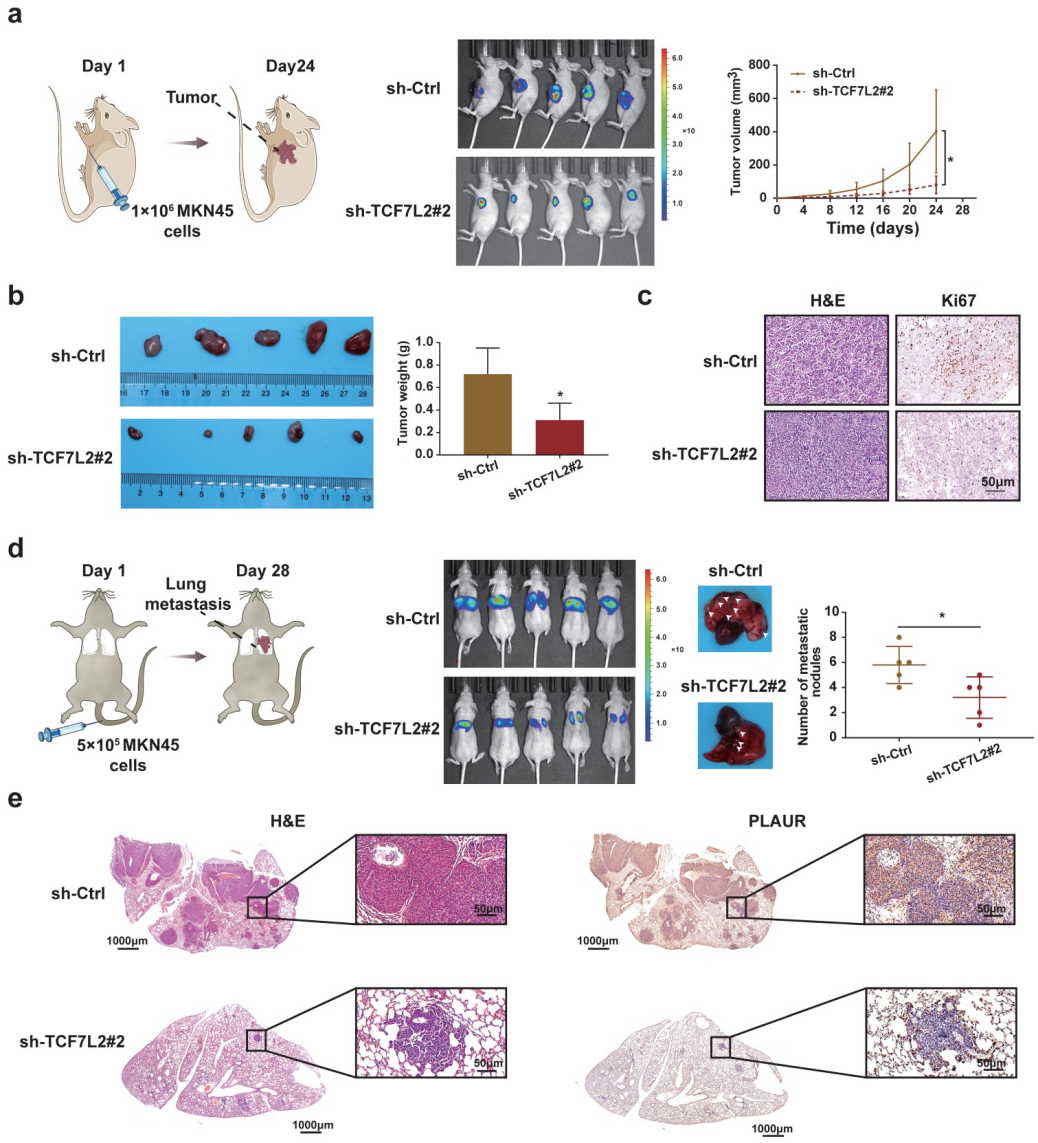
** TCF7L2 promotes the GC process *in vivo*. a** Knockdown of TCF7L2 inhibited the growth of subcutaneous xenograft tumors in nude mice. Bioluminescence images of the nude mice subcutaneous tumors were displayed through a small animal* in vivo* imaging system, and the tumor growth curve was measured for 24 days. **b** Gross specimen and average weight of the tumor after animals were sacrificed. Knockdown of TCF7L2 inhibited the volume and weight of subcutaneous xenograft tumors in nude mice. **c** Histological characteristics and Ki67 expression of the tumor were examined via H&E staining and IHC, respectively. Knockdown of TCF7L2 inhibited the Ki67 expression in subcutaneous xenograft tumors. **d** Knockdown of TCF7L2 inhibited lung metastasis. The bioluminescence image of lung metastases and the count of lung metastatic nodules was obtained 28 days after tail veins were injected with GC cells. The white arrows represent the lung metastasis nodule. **e** Lung metastases were confirmed via H&E staining (left); knockdown of TCF7L2 inhibited PLAUR expression in lung metastases evaluated by IHC (right). Compared with the control group, * *P* < 0.001.

To clarify the functions of TCF7L2* in vivo*, we established nude mouse models of subcutaneous tumor and lung metastasis by injecting MKN45 cells, according to previous reports. As shown in **Figure 7a**, when MKN45 cells with sh-Ctrl or sh-TCF7L2#2 were injected, the fluorescence signal intensity and tumor growth rate of the subcutaneous tumors in the sh-Ctrl group were significantly higher than those in the sh-TCF7L2#2 group. The weight of the tumors was calculated after euthanizing the mice. The sh-Ctrl group exhibited significantly heavier tumors than the sh-TCF7L2#2 group (**Figure 7b**). We also identified tumors via H&E staining. IHC results indicated weaker tumor tissue expression of Ki67 in the sh-TCF7L2 group than in the sh-Ctrl group (**Figure 7c**). On the 28^th^ day following tail vein injection of MKN45 cells, the fluorescence signal intensity in the lung from metastatic tumors was significantly higher in the sh-Ctrl group than that in the sh-TCF7L2 group. We also observed fewer average numbers of nodules on the lung surfaces for mice in the sh-TCF7L2 group than those in the sh-Ctrl group (**Figure 7d**). Importantly, IHC staining revealed that the knockdown of TCF7L2 inhibited PLAUR expression in lung metastasis nodules of the mice (**Figure 7e**). In summary, TCF7L2 promoted the growth and metastasis of GC as well as regulated the expression of PLAUR *in vivo*.

### Role of PLAUR in TCF7L2 induction of GC proliferation, anoikis resistance, and migration

**Figure 8:**
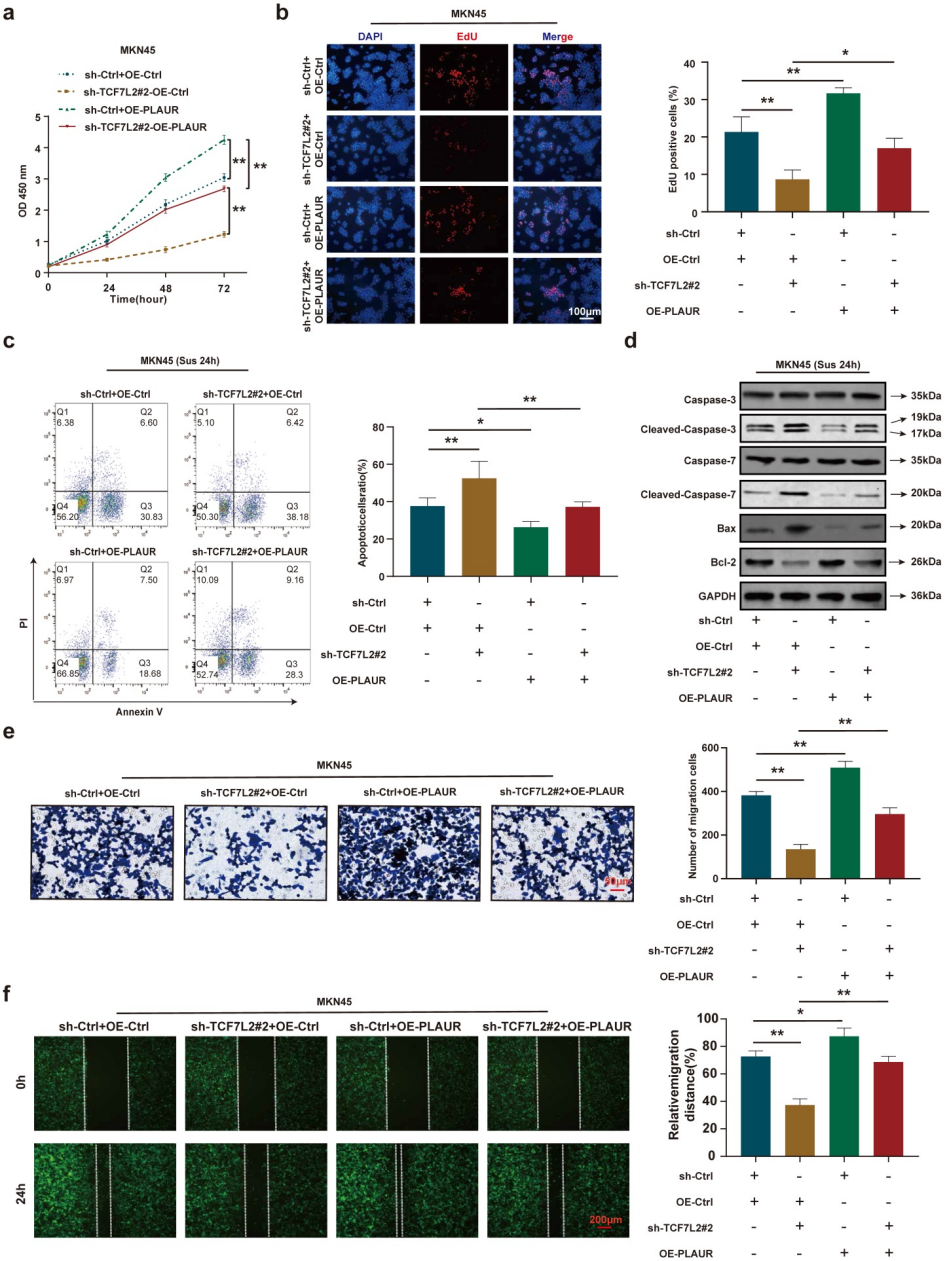
** PLAUR is essential for the malignant phenotype of GC cells mediated by TCF7L2. a-b** Cell proliferation of MKN45 cells with knockdown of TCF7L2 or control, and re-expression of PLAUR in TCF7L2 depleted cells, as quantified by CCK-8 **(a)** and EdU** (b)** assay, respectively. **c** After GC cells were suspended for 24 h, the anoikis resistance of MKN45 cells with knockdown of TCF7L2 or control and re-expression of PLAUR in TCF7L2 depleted cells were assessed via flow cytometry. **d** After GC cells were suspended for 24 h, western blot for apoptosis-related proteins in MKN45 cells with the depletion of TCF7L2 or control, or re-expression of PLAUR in TCF7L2 depleted cells. **e-f** Measurement of cell migration by transwell** (e)** and wound healing** (f)** assays using MKN45 cells with depletion of TCF7L2 or control, or re-expression of PLAUR in TCF7L2 depleted cells. * *P*<0.05, ** *P*<0.001.

To further validate that PLAUR is a downstream target gene of TCF7L2, we again performed rescue experiments using sh-TCF7L2 MKN45 cells to overexpress PLAUR and observed the changes in proliferation, anoikis resistance, and cell migration. CCK8 and EdU assays demonstrated that TCF7L2 knockdown inhibited the proliferation of MKN45 cells, and the reduction of MKN45 cell proliferation by sh-TCF7L2 was reversed by co-transfection with PLAUR (**Figure 8a-b**). As shown in **Figure 8c**, the knockdown of TCF7L2 inhibited the anoikis resistance of MKN45 cells, which was partially reversed by co-transfection with PLAUR. Western blot revealed that when MKN45 cells were suspended for 24 h, the sh-TCF7L2-induced increases in cleaved-caspase-3/7 and Bax expression were partially reversed by the overexpression of PLAUR, and the sh-TCF7L2-induced decrease in Bcl-2 expression was also partially reversed by PLAUR overexpression (**Figure 8d**). In transwell and wound healing assays, TCF7L2 knockdown inhibited the migration capacity of MKN45 cells, and the sh-TCF7L2-induced reduction of this migration capacity was partially rescued by co-transfection with PLAUR (**Figure 8e-f**). These results indicated that TCF7L2 stimulates the proliferation, anoikis resistance, and migration of GC cells in a PLAUR-dependent manner.

### TCF7L2 is associated with poor prognosis of patients with GC

**Figure 9:**
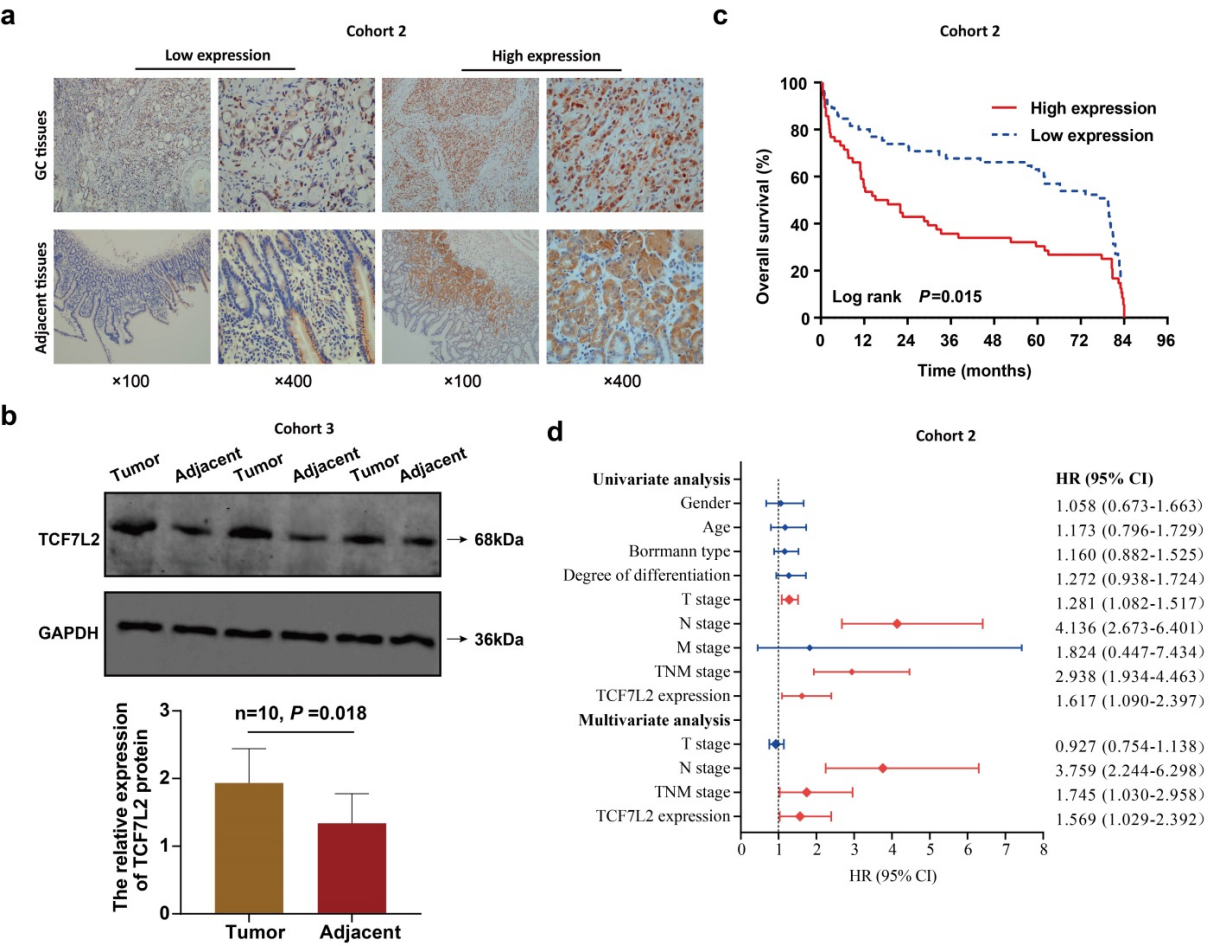
** High TCF7L2 expression predicts poor prognosis of patients with GC. a** Typical images of TCF7L2 protein in GC and adjacent tissues stained by IHC. In GC tissues, TCF7L2 protein was located in the nucleus and cytoplasm; in adjacent tissues, TCF7L2 protein was primarily located in cytoplasm. **b** Survival curve of 121 GC patients (cohort 2). High TCF7L2 expression correlated with poor prognosis of GC patients.** c** TCF7L2 protein was quantitatively analyzed via western blot in 10 pairs of fresh GC and adjacent tissues (cohort 3). **d** Independent prognostic factor for overall survival of 121 patients with GC, shown as a forest plot.

Finally, this study assessed the effects of TCF7L2 on the prognosis of patients with GC. TCF7L2 protein expression in 121 pairs of GC and adjacent tissues (cohort 2) was examined via IHC. As shown in** Figure 9a**, TCF7L2 protein was stained to varying degrees in both the cell cytoplasm and nucleus. Notably, TCF7L2 protein was primarily localized in the cell nuclei of GC tissue, as well as in the cytoplasm in adjacent tissues. This suggested that TCF7L2 exerts a cancer-promoting role in the nucleus of GC cells. A high expression of TCF7L2 was observed in 46.3% (56/121) of GC tissues, while showing a significant difference (χ^2^ = 6.305, *P* = 0.012) in only 30.6% (37/121) of adjacent tissues. Meanwhile, we quantitatively explored the expression levels of TCF7L2 protein in 10 pairs of fresh GC and adjacent tissues (cohort 3) via western blot. As shown in **Figure 9b**, TCF7L2 protein was more highly expressed in GC tissues than in adjacent tissues (*P* < 0.001). Consistent with prior bioinformatics results, TCF7L2 appeared to be upregulated in GC tissues.

For the analysis of the correlation between TCF7L2 and patient prognosis, we divided the 121 patients with GC into TCF7L2 “high expression” (n = 56) and “low expression” (n = 65) groups. Meanwhile, we collected and statistically analyzed the clinicopathological data of all patients with GC and found that high TCF7L2 expression was closely linked to lymph node metastasis and TNM staging (*P* < 0.05); however, it was not linked to such clinicopathological features such as gender, age, tumor size, Borrmann classification, tumor location, and degree of differentiation (*P* > 0.05) (**Table 1**). Follow-up analyses revealed that the survival time of patients with GC with low TCF7L2 expression was markedly longer than those with high TCF7L2 expression (**Figure 9c**). Finally, we established a Cox regression model by utilizing the clinicopathological and survival data of 121 patients with GC to assess whether TCF7L2 could act as an independent risk predictor for the prognosis of patients with GC. Univariate analysis revealed that deeper tumor invasion (T stage), lymph node metastasis (N stage), advanced TNM stage, and high TCF7L2 expression were significantly correlated with a poor prognosis for patients with GC (**Figure 9d**). According to the multivariate analysis, high TCF7L2 expression could serve as an independent risk predictor for poor prognosis of patients with GC (*P* = 0.044).

## Discussion

This study revealed that TCF7L2 was highly expressed in GC, which is closely associated with the poor clinicopathological features of patients. High TCF7L2 expression is an independent risk factor for poor prognosis in patients with GC. *In vitro*, we confirmed that TCF7L2 promotes the proliferation, anoikis resistance, and migration of GC cells. *In vivo*, we found that TCF7L2 promoted tumor growth and metastasis in nude mice. Importantly, TCF7L2 was found to be a major transcriptional regulator of *PLAUR*, with binding sites within the promoter region of *PLAUR*, leading to its transcriptional activation. Similarly, we also verified that PLAUR promotes the malignant behaviors of GC *in vitro* and* in vivo*, particularly the anoikis resistance of GC cells. Altogether, our data revealed that the transcription factor TCF7L2 is overexpressed and abnormally activates *PLAUR* in GC and is involved in promoting the anoikis resistance and migration of GC cells.

Members of the fibrinolytic system play important roles in cellular chemotaxis, invasion, migration, regulation of various cytokines and growth factors, immune response, and angiogenesis 13, 14, 43, 44. PLAUR, as one of the major members of this family, also plays important roles in the malignant biological process of GC, particularly the migration of GC cells. It has been established that the collagen fibrin network is formed in the intercellular space via stromal cell secretion, which effectively prevents the migration of tumor cells, owing to its intricate structure 45, 46. Tumor cells destroy the aforementioned stromal network by activating the fibrinolytic system through PLAUR, thereby creating favorable conditions for metastasis. Through semi-quantitative IHC, Heiss et al. 47 revealed that PLAUR expression was increased in GC tissues. Based on Cox analysis, they further found that PLAUR was significantly negatively correlated with the survival time of patients with GC. Additionally, many other studies have confirmed that PLAUR is highly expressed in GC, exhibiting a close association with the distant metastasis of tumors 48-50. Thus, PLAUR is an important regulator of GC cell migration. In this study, we verified that PLAUR promotes the malignant process of GC in both *in vitro* and *in vivo* experiments. Notably, to the best of our knowledge, there have been no previous reports on the correlation between PLAUR and anoikis resistance of GC cells. Nevertheless, in cases like melanoma and prostatic cancer, researchers have proposed a relationship between PLAUR and anoikis resistance 51-53. We used an anoikis model with GC cells suspended for 24 h and further validated the ability of PLAUR to promote the anoikis resistance of GC cells.

Furthermore, this study explored the molecular mechanism whereby PLAUR is upregulated in GC. We analyzed the reasons for the high PLAUR expression from an abnormal transcription perspective, identifying TCF7L2 as a potential transcriptional regulator of PLAUR. We found that TCF7L2 protein was localized primarily in the nuclei of GC cells. Thereafter, through ChIP-qPCR and dual luciferase reporter assays, we not only confirmed the presence of binding sites between TCF7L2 and *PLAUR* promoter region, but also verified that TCF7L2 positively regulated PLAUR expression in GC via transcription. PLAUR is also regulated by other transcription factors. For example, FOXM1c is upregulated in pancreatic cancer which, as a transcription factor, has binding sites within the *PLAUR* promoter and causes the upregulation of PLAUR, ultimately affecting the migration of pancreatic cancer cells 57. Based on luciferase assays, researchers found that the hypoxia-induced overexpression of HIF-1α activated the transcriptional activity of the *PLAUR* promoter, thereby enhancing its expression in cervical cancer, so that the cervical cancer cells were more invasive under hypoxic conditions 58. In hepatoma cells, ETV4 directly binds to the core region of the *PLAUR* promoter, while PBK can enhance the binding between ETV4 and the *PLAUR* promoter 59. However, the role of TCF7L2 in GC remained unclear, particularly its correlation with the anoikis resistance of GC cells.

TCF7L2, as a downstream effector of Wnt/β-catenin signaling pathway, contains a highly conserved HMG box and a small basic peptide motif, similar to other members of its family 60, 61. Together, these two structures constitute the HMG DNA domain, which can recognize specific DNA sequences 60, 62, 63. Additionally, the members of this family also contain a β-catenin binding domain and a C-clamp domain 60, 64, 65. The C-clamp domain is located at the carboxyl terminal of the HMG DNA domain and has a specific DNA binding activity 60, 66. Thus, TCF7L2 has two specific DNA binding domains. In various solid tumors, TCF7L2 is abnormally expressed and is involved in the malignant process. Compared to low-grade gliomas, TCF7L2 is upregulated in glioblastoma tissues, exhibiting a close association with the poor prognosis of patients with glioblastoma 67. In pancreatic cancer, high TCF7L2 expression predicts a poor prognosis. TCF7L2 leads to the upregulation of HIF-1α by inhibiting the expression of EGLN2, an Egl-9 family member, thereby positively regulating aerobic glycolysis 68. As a transcriptional activator of *WNT7B*, TCF7L2 binds to its promoter region to further facilitate the growth and migration of GC cells 69. The long non-coding RNA SNHG11 can enhance the oncogenic autophagy of GC cells, thereby promoting the proliferation, stemness, migration, invasion, and epithelial-mesenchymal transition of tumor cells. Its upregulation in GC is achieved by transcriptional activation of TCF7L2 70. Our study revealed that TCF7L2 is upregulated in GC tissues and cells. Through *in vitro* cell experimentation, we verified that TCF7L2 promotes the proliferation, anoikis resistance, and migration capacity of GC cells. In our *in vivo* experiments, TCF7L2 promoted tumor growth and metastasis in nude mice. Notably, anoikis resistance is a key pre-step for distant tumor metastasis, and TCF7L2 was closely associated with the anoikis resistance of GC cells. Through functional rescue experiments, this study also confirmed that TCF7L2 plays an important role in the malignant process of GC in a PLAUR-dependent manner.

## Conclusions

**Figure 10:**
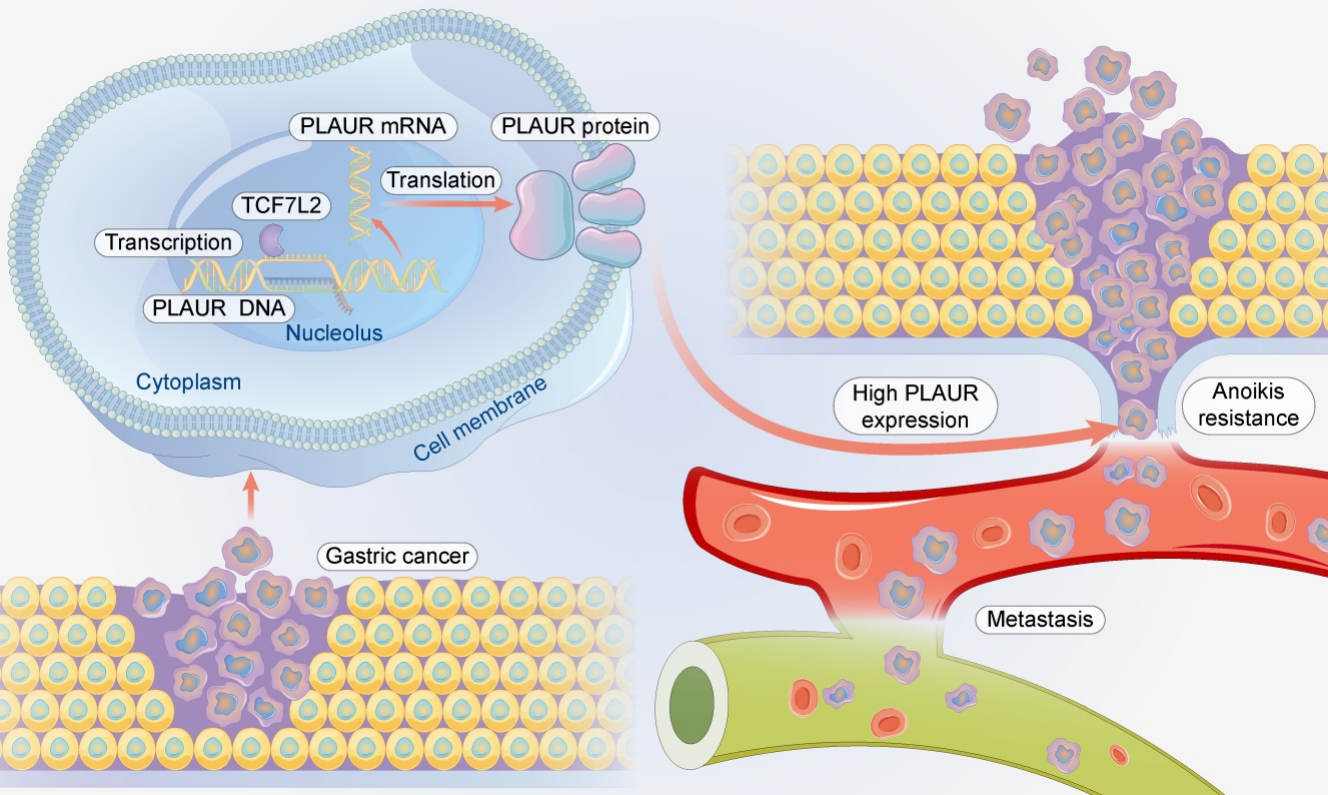
** Schematic summary.** Mechanistically, TCF7L2 is upregulated in GC and transcriptionally activates PLAUR, which promotes anoikis resistance and metastasis of GC cells.

In conclusion, TCF7L2 is highly expressed in GC and is an independent risk predictor for the poor prognosis of patients with GC. TCF7L2 enhances the proliferation, anoikis resistance, and migration of GC cells in a PLAUR-dependent manner (**Figure 10**). Our study clarifies that PLAUR affects the upstream molecular regulation of GC, which also provides a theoretical basis for the use of TCF7L2 and PLAUR as potential therapeutic targets for GC.

## Supplementary Material

Supplementary figures and tables 1, 2, 3, 4, 5, and 7.

Supplementary table 6.

## Figures and Tables

**Table 1:** The relationship between TCF7L2 expression and clinicopathological characteristics of patients with GC

Factor	TCF7L2 expression		*χ* ^2^	*P*-value
	Low (*n*=65)	High (*n*=56)		
**Gender**			2.690	0.101
Male	45 (69.2)	46 (82.1)		
Female	20 (30.8)	10 (17.9)		
**Age**			0.132	0.716
≤60y	35 (53.8)	32 (57.1)		
>60y	30 (46.2)	24 (42.9)		
**Tumor size**			0.963	0.326
≤5cm	45 (69.2)	34 (60.7)		
>5cm	20 (30.8)	22 (39.3)		
**Borrmann type**			1.413	0.703
I	9 (13.8)	4 (7.1)		
II	13 (20)	12 (21.4)		
III	42 (64.6)	39 (69.6)		
IV	1 (1.5)	1 (1.8)		
**Type of gastrectomy**			2.354	0.671
Gastrectomy-partial	10 (15.4)	10 (17.9)		
Near-total gastrectomy	34 (52.3)	22 (39.3)		
Total gastrectomy	18 (27.7)	20 (35.7)		
Gastrectomy with esophagus	1 (1.5)	2 (3.6)		
Gastrectomy with other organs	2 (3.1)	2 (3.6)		
**Degree of differentiation**			1.644	0.439
Well	3 (4.6)	6 (10.7)		
Moderate	24 (36.9)	20 (35.7)		
Poor	38 (58.5)	30 (53.6)		
**T stage**			2.478	0.649
Tis	1 (1.5)	3 (5.4)		
T1	6 (9.2)	7 (12.5)		
T2	9 (13.8)	7 (12.5)		
T3	8 (12.3)	4 (7.1)		
T4	41 (63.1)	35 (62.5)		
**N stage**			4.447	0.035
N0	32 (49.2)	17 (30.4)		
N1-N3	33 (50.8)	39 (69.6)		
**M stage**			0.011	0.915
M0	64 (98.5)	55 (98.2)		
M1	1 (1.5)	1 (1.8)		
**TNM stage**			4.682	0.030
I-II	36 (55.4)	20 (35.7)		
III-IV	29 (44.6)	36 (64.3)		

## Abbreviations

ChIP: Chromatin-immunoprecipitation
co-IP: Co-immunoprecipitation
DAPI: 4',6-diamidino-2-phenylindole
EdU: 5-ethynyl-2'-deoxyuridine
GC: gastric cancer
IHC: immunohistochemistry
PLAUR: urokinase-type plasminogen activator receptor
TCF7L2: transcription Factor 7 Like 2
HMG: high-mobility group
FBS: fetal bovine serum
shRNA: small hairpin RNA
qRTPCR: quantitative reverse transcription polymerase chain reaction
PVDF: polyvinylidene difluoride
IF: immunofluorescence
LC-MS/MS: liquid chromatography tandem mass spectrometry
H&E: hematoxylin and eosin
IHC: immunohistochemistry
RPMI: Roswell Park Memorial Institute
RIPA: radio immunoprecipitation assay
STAD: stomach adenocarcinoma
