# Supplementary material for: TCF7L2 promotes anoikis resistance and metastasis of gastric cancer by transcriptionally activating PLAUR
Source: Int J Biol Sci. 2022 Jul 11;18(11):4560–77. doi: 10.7150/ijbs.69933 (PMC9295057; doi:10.7150/ijbs.69933)
Supplement: Supplementary file 1 — Supplementary figures and tables 1, 2, 3, 4, 5, and 7. [file ijbsv18p4560s1.pdf]

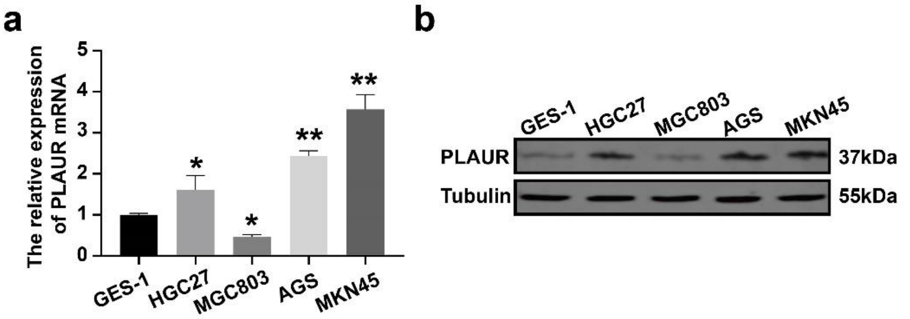

Figure S1 The expression of PLAUR mRNA and protein in GES-1 and GC cell lines which were detected by qRT-PCR **(a)** and western blot **(b)**, respectively.

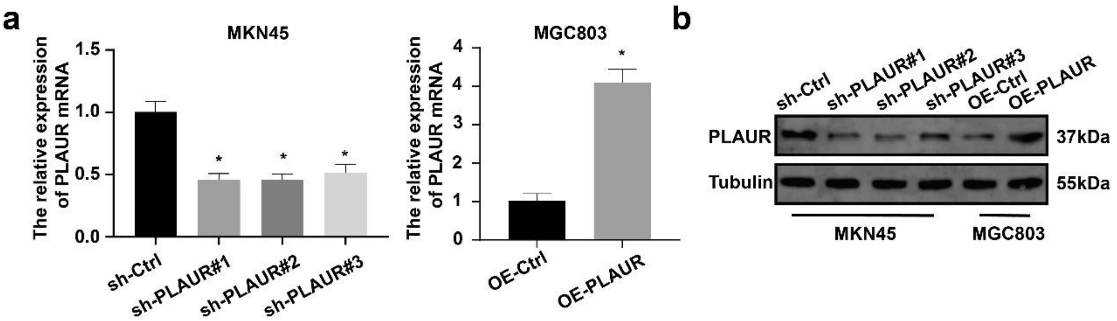

Figure S2 The inhibition rate of PLAUR in MKN45 cells and the overexpression rate in MGC803 cells were verified by RT-qPCR **(a)** and western blot **(b)**, respectively.

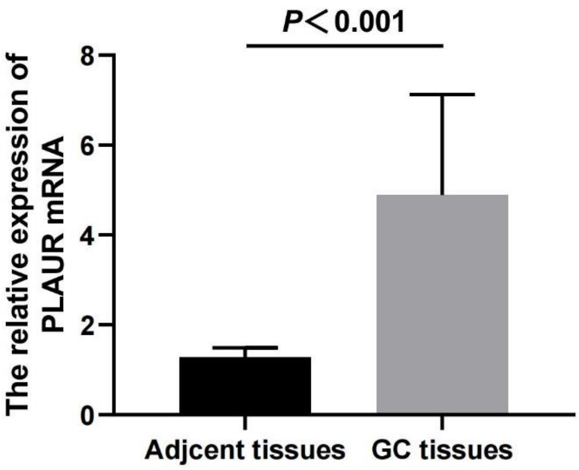

Figure S3 Detection of PLAUR mRNA expression in 15 pairs of GC and corresponding adjacent tissues by RT-qPCR.

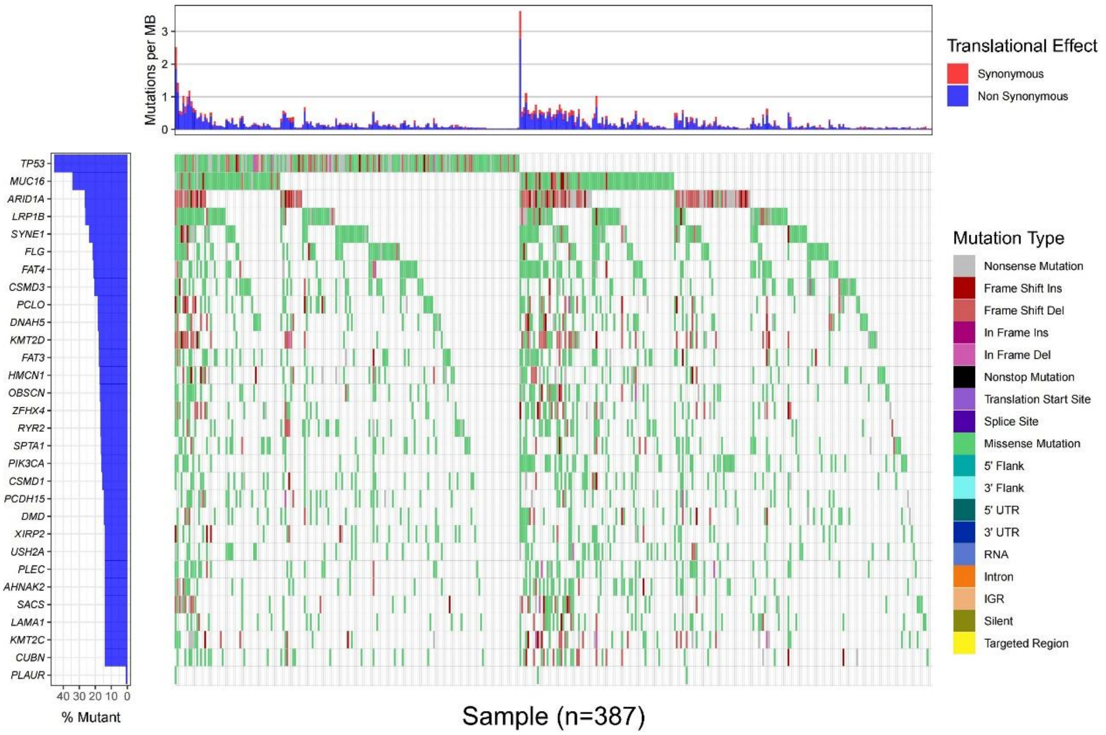

Figure S4 Waterfall chart of mutant genes in STAD which is based on TCGA-STAD-seq.

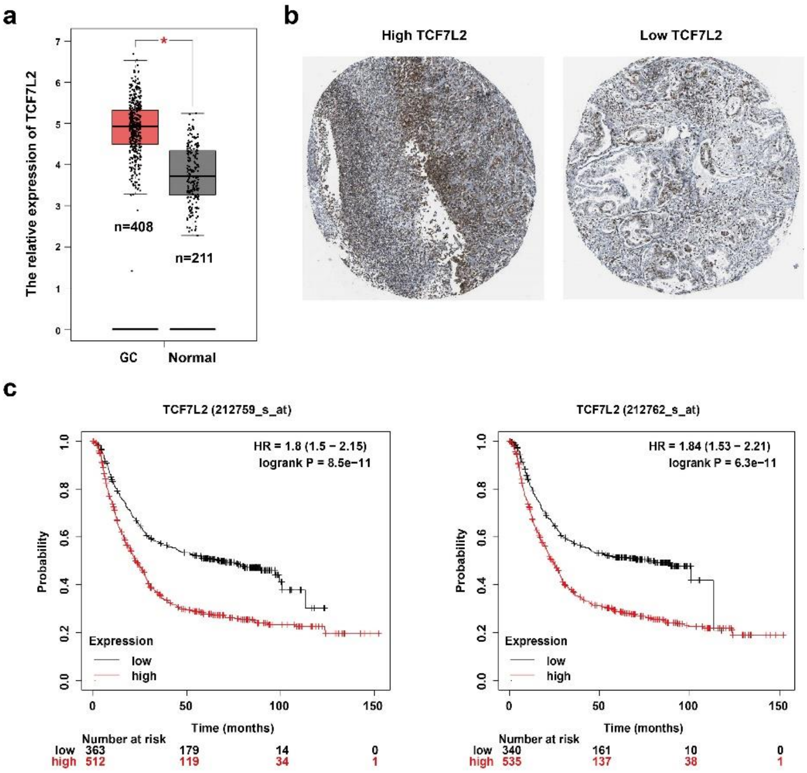

Figure S5 The relationship between TCF7L2 and GC is analyzed based on bioinformatics. **(a)** GEPIA analyzed the expression of TCF7L2 in GC and normal samples. **(b)** The localization of TCF7L2 protein in GC tissue was observed by HPA. **(c)** The relationship between TCF7L2 and survival time of patients with GC which was evaluated by KM Plotter.

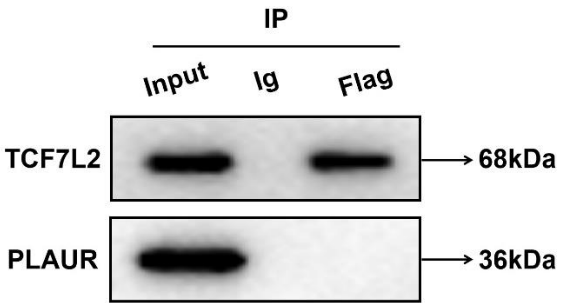

Figure S6 Co-IP analysis shown that there is no interaction between TCF7L2 and PLAUR protein.

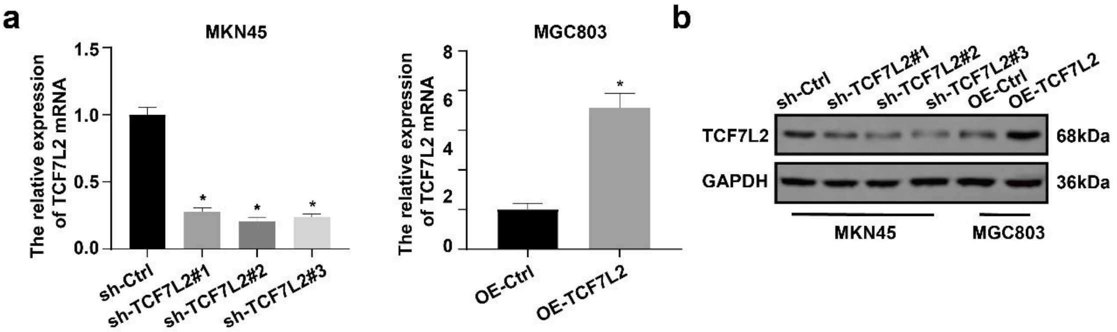

Figure S7 The inhibition rate of TCF7L2 in MKN45 cells and the overexpression rate in MGC803 cells were verified by RT-qPCR **(a)** and western blot **(b)**, respectively.

Supplementary Table S1 The target sequences of shRNAs

Gene name	Sequences (5’-3’)
PLAUR #1	CCACTTCCTGAAATGCTGCA
PLAUR #2	CACTCTCCTCTGGACCTAAA
PLAUR #3	GCCGTTACCTCGAATGCATT
TCF7L2 #1	CCTTTCACTTCCTCCGATTA
TCF7L2 #2	CCCACATAAAGAAACCTCTT
TCF7L2 #3	AGAGAAGAGCAAGCGAAATA

Supplementary Table S2 Primer information for qRT-PCR

Gene name	Forward primer	Reverse primer
PLAUR	GGTGACGCCTTCAGCATGA	CCCACTGCGGTACTGGACAT
TCF7L2	AGAAACGAATCAAAACAGCTCCT	CGGGATTTGTCTCGGAAACTT
GAPDH	AGGTTGTCTCCTGCGACTTCA	GGGTGGTCCAGGGTTTCTTAC

Supplementary Table S3 The information of primary antibodies

Antibody name	Manufacturer	Article Number
Anti-PLAUR	Santa Cruz	sc-13522
Anti-TCF7L2	Cell Signaling Technology	#2569
Anti-TCF7L2 (IP)	Proteintech	13838-1-AP
Anti-TCF7L2 (IF)	Bioss	bsm-52543R
Anti-Ki67	Proteintech	27309-1-AP
Anti-Caspase-3	Cell Signaling Technology	#9662
Anti-Cleaved Caspase-3	Cell Signaling Technology	#9661
Anti-Caspase-7	Cell Signaling Technology	#12827
Anti-Cleaved Caspase-7	Cell Signaling Technology	#9491
Anti-bax	Cell Signaling Technology	#2774
Anti-bcl-2	Cell Signaling Technology	#15071
GAPDH	Proteintech	60004-1-Ig
α-Tubulin	Proteintech	66031-1-Ig

Supplementary Table S4 The information of *PLAUR* promoter sequence

PLAUR promoter sequence
GGGAACTTTGCTTTGCGTCCTGCTGTGCCCCTAGCTCCTGGACCAGTGCCCAGCACAAAGTAGGTC<br>CTTGGTAGATGTTAAAGGAATGTGTTGAGCCCTCCATTTCGGCCAGCAGCTATTCCAGGCATTGCAC<br>CTACATTAAATTGCTCTGTTTAGTTAGATGCATCCTAAGACGGGGAGACAATTTTATCTTTAGTATTTT<br>ATTTTACTTAAAAAAAAAAAAAACTTGGCCAGGTGCAGTGGCTCATGCCTGTAATCCCAGCACTTT<br>GGAAGGACAAGGCAGGTGGATCACCTGAGGTCAGGAGTTCGAGACCACCCTGACCAACATGGTG<br>AAACCCCATCTCTACTAAAAATACAAAAATTAGCCGGGTGTGCTGGCGTGCCCCTGTAATCCCAGTT<br>ACTCAGGAGGCTGAGGCAGGAGAATTGCATGAACCCAGGAGGCGGAGATTGCAGTGGCCGAGATT<br>GCACCACTGCACTCTAGCCTGGGTGACAGAGTGAGACTTCGTCTCAATAAATAAATAAATAAATAAT<br>AAAAAGAAAGAAAAAACTTATTATAAGCCTTTTCAAACCCACAGAAAAGGAGACAGAATGGCATA<br>ATATGCCCTTATATACTGATTACCTTTATATAATTGATACAACCATTTGGCTATTTTGCCTTTATTTTCTA<br>CTCAAGTATTTATTTATTTATTATTTTTTTGAGACAGGGTCTCGCTCTGTTGCCCAGGTTGGAGTGCA<br>GTGGTGCAATCATAGCTCACTGCAGCTTCCAGTTCCCAGGTTCAAGCGGTCCTCCCACCTTAGCCTC<br>CCAACTACCTAGGACTACAAGTACGGGCCACTGCACCCAGCTAATTTTATTTTATTTTTAATTTCTTT<br>TGGAAAGATGAGGGCTCTTTATGTTTCCCAAGCTGATCTTGAACTCCTGGCCCCAAGTGATCCTCCT<br>GCCTTGGCTTCCCAAAGCATTGGTGTTACAGGCATGAGCCACCTCATCTGACCTCTTCTAAAATATT<br>ATAAAATAAATACAGACATCAAAATGAAAACAAAAGAAAAAGAAAAAACAAAACAAAAATCCCCA<br>AAACAACAACAACAAAAAATGCAGACATCAATAACATTCACCACTAAATACTCTAGTAAGCATCTG<br>CAAAAACAAACAAACAAACAAAAACCAGAATCCCACCTAAGCATACAGCCATTGTCGTAACAGTG<br>ATATCTACTTTTTTGGCTGAAGTGTCTTTTTTTTTTTTTTTTTTTTTTCTGTGAGAAGGAGTCTTGTT<br>CTGTTGCCAGGCTGGAGTGCAGTGGCACGATCTCGGCTCACTGCAACCCAACCTCCGCCTCCCGGG<br>TTCAAGCGATTCTCCTGCCTCAGCCTCCTAAGTAACACACACACGCCACTACGCCCGGCTAATTTTT<br>GTATTTTTAGTAGAGACGGGGTTTCACCATTAAGTGTTTTAATGTAGGTGCAATGCCTGGAATAGCTG<br>CTGGCCAAAATGGAGGGCTCAACACATTCCTTTAACATTTACCAAGGACCTACTTCGTGCTGGGCA<br>CTGGTCCAGGAGCTGGGGGCACAGCGGGAAGCAAAGCAAGGGTTAAGTGTTTTAAGTGTTTAAGA<br>GATGCATATTTCAGGATGCATCTCTAGATAAGGACATTTTCCAAAATACCAGTATCCCTCCTGACAAA<br>ACTAACAAAAATCCTGTTAGCCAAATAATCAGCCACATTCATATTTACCGTCAAAGTTTTTATCCTCA<br>TTTTACAGCAGTGGAGAGCGATTGCCCCGGGTCCCACGTTAGGAAGAGAGAGAACTGGGATTTGC<br>ACCCAGGCAATCTGGGGACAGAGCTGTGATCACAACTCCATGAGTCAGGGCCGAGCCAGCCCCTT<br>CACCACCAGCCGGCCGCGCCCCGGGAAGGGAAGTTTGTGGCGGAGGAGGTTCGTACGGGAGGAG<br>GGGGAGGCGCCCACGCATCTGGGGCTGACTCGCTCTTTCGCAAAACGTCTGGGAGGAGTCCCTGG<br>GGCCA

Supplementary Table S5 The information of primer sequences

Sequence name	Forward primer	Reverse primer
Seq1	GCAGACATCAATAACATTCACCACT	GCAACAGAACAAGACTCCTTCTCA
Seq2	CCACCTCATCTGACCTCTTCTAAA	TTGTTTGTTTGTTTTTGCAGATGCT
Seq3	AAACCAGAATCCCACCTAAGCATAC	CCAGCCTGGCAACAGAACA
Seq4	GAGGGCTCTTTATGTTTCCCA	GGGGATTTTTGTTTTGTTTTTTCTT
Seq5	AGGACTACAAGTACGGGCCA	ATGCCTGTAACACCAATGCTTT

Supplementary Table S7 *PLAUR* promoter site capable of binding to TCF7L2 (top 5 for scoring)

MatrixID	Name	Score	Relative score	Start	End	Strand	Sequence
MA0523.1	TCF7L2	11.4503	0.900683	1018	1031	+	agacatcaaaatga
MA0523.1	TCF7L2	7.23193	0.842383	1076	1089	+	caacaacaaaaaat
MA0523.1	TCF7L2	5.59419	0.819749	206	219	+	ttacttaaaaaaaa
MA0523.1	TCF7L2	5.51573	0.818665	531	544	+	taaaaagaaagaaa
MA0523.1	TCF7L2	5.08067	0.812652	1092	1105	+	agacatcaataaca
